# Hypothalamus, Neuropeptides and Socioemotional Behavior

**DOI:** 10.3390/brainsci13091303

**Published:** 2023-09-11

**Authors:** Andrea Caria

**Affiliations:** Department of Psychology and Cognitive Sciences, University of Trento, 38068 Rovereto, Italy; andrea.caria@unitn.it

A large body of evidence from old stimulation and lesion studies on the hypothalamus in animals and humans demonstrates that this subcortical area significantly affects socioemotional behavior [1,2,3,4]; more recent optogenetic studies extended this evidence by showing that the stimulation of distinct hypothalamic nuclei elicits defensive and aggressive responses [5,6,7,8,9,10,11]. Additional studies have revealed that hypothalamic stimulation can also trigger pleasant responses and prosocial behavior [12,13,14,15]. Similarly, studies on the effects of the intranasal administration of oxytocin (OXT) and arginine-vasopressin [16,17,18,19,20,21,22]—two evolutionarily conserved hypothalamic peptides—have reported heterogenous and divergent results, including augmented prosocial attitudes and behaviors as well as increased mistrust, competition, and aggressive reactions [23,24,25,26].

Notwithstanding these findings on the important hypothalamic role in mediating socioemotional responses, this brain region was, for a long time, mainly considered a relay station, passing signals among the amygdala, basal forebrain, and mesencephalic structures to support behavioral, autonomic, and endocrine components of higher-level controlled emotional responses. Furthermore, until recently a rather limited number of investigations explored, in humans, the direct involvement of the hypothalamus during socioemotional responses. In addition, functional neuroimaging studies often undervalued or neglected the hypothalamic contribution to social cognition and behavior.

Building on this evidence, this Special Issue, titled “Hypothalamus, Neuropeptides and Socioemotional Behavior”, attempted to collect novel scientific reports highlighting the central role of the hypothalamus in socioemotional behavior from different neurobiological perspectives and methodologies in both healthy and clinical human populations, and in animals. The studies hosted in this Special Issue, including neuroimaging or neurochemical investigations, indeed provided some additional insights into the relevance of the hypothalamus and its associated neuropeptides in modulating socioaffective responses.

For instance, considering the well-known modulatory role of oxytocin in social interactions, including social comparisons and intergroup competition, the research of Kim and colleagues [27] examined whether and how OXT differentially influences social comparisons in an intergroup situation. Using a double-blind placebo-controlled design, they studied the effects of intranasal OXT administration on participants performing a social comparison task, playing a gamble-like card selection game with either an in-group or out-group member. They reported that the OXT-treated participants showed a greater social comparison effect in games with an out-group member than in games with an in-group member. Specifically, the participants in the OXT treatment condition showed a greater acceptance rate for relative gain and a lower acceptance rate for relative loss while playing with an out-group member rather than an in-group member. In contrast, no such effect was observed among placebo-treated participants. These findings indicated that OXT also modulates intergroup social comparisons with out-group versus in-group members.

Linking OXT and parental behavior, Cataldo and colleagues [28] aimed to extend our current knowledge of the interactions between oxytocin receptor gene (OXTR) polymorphism, parental attachment, and socioemotional responses. Their research investigated the influence of parental bonding and genetic allelic variation in an OXTR polymorphism (rs53576)—an allelic variation that has been associated with socioemotional disorders such as autism [29]—on the levels of anxiety and avoidance in adult relationships. In 313 young adults belonging to two different cultural contexts, namely Italy and Singapore, they observed main effects of maternal characteristics, care, and overprotection on the levels of experienced anxiety and avoidance. In addition, they reported an interaction effect between OXTR rs53576 and maternal overprotection in explaining levels of anxiety and avoidance, suggesting differential environmental susceptibility between Western and Eastern groups despite equivalent individual genetic features.

Aiming to explore potential group differences in endogenous OXT concentrations between individuals with autism spectrum disorder, ASD, and neurotypical (NT) controls, Moerkerke and colleagues [30] conducted a meta-analysis of studies showing correlations between individual differences in endogenous OT levels and social deficits, thus suggesting a role of endogenous OT in the pathogenesis of social impairments characterizing ASD. An analysis of 18 studies including 1422 participants revealed that endogenous OXT levels were lower in children with ASD as compared to NT controls, but not in adolescent and adult populations. Additionally, while no significant subgroup differences were found in regard to sex, the group difference in the OXT levels of individuals with versus without ASD appeared only in studies with male participants and not female participants. These results suggest that atypical development might possibly be coupled with developmental changes in endogenous OT levels; however, further research adopting more consistent and appropriate methodologies is still necessary to confirm this assumption.

In addition to the well-established relationship between oxytocin and social behavior, phoenixin, a novel peptide that has been associated with reproductive functions in both the hypothalamus and pituitary [31], has recently attracted attention. Friedrich and colleagues’ study [32] investigated, in rats, whether phoenixin could play a role in the response to inflammatory stress. They reported that lipopolysaccharide-induced inflammatory stress was associated with phoenixin-immunoreactive brain nuclei including the central amygdaloid, supraoptic nucleus, arcuate nucleus, bed nucleus of the stria terminalis, and the medial part of the nucleus of the solitary tract. These results extended previous findings that indicated distinct changes in neuronal activity and immunoreactivity in relation to emotional stressor restraints [33]. Considering the neuronal structures involved, these findings further suggest that phoenixin might impact emotional-related stressful responses through its influence on several subcortical socioemotional brain centers.

In relation to hypothalamic nuclei, Carollo and colleagues [34] reviewed the current literature using a scientometric approach to examine the relationship between the medial preoptic area (MPOA) and parental behavior. They observed that current studies, mainly on rodents, focused on the properties of the MPOA as well as on the interactions of the MPOA with other brain networks, such as the reward circuits, in response to maternal behavior. However, more recent studies on the MPOA focused on human populations and also considered paternal behavior.

Finally, Caria and Dall’Ò [35] provided a synthesis of human neuroimaging studies reporting hypothalamic activation during affiliative, cooperative interactions, ticklish laughter and humor, and during aggressive as well as antisocial interactions. Their systematic review revealed a growing number of investigations showing that the evolutionarily-conserved hypothalamic neural circuity substantially contributes to multiple and diverse aspects of human socioaffective behavior. All of these distinct behavioral responses appear to be regulated through widespread functional interactions of the hypothalamus with multiple cortical and subcortical regions [36].

On the basis of the observed heterogeneity of hypothalamus-mediated socioemotional responses, I propose that the hypothalamus and its associated peptides might play an extended functional role in species survival and preservation, ranging from exploratory and approaching behaviors, promoting social interactions, to aggressive and avoidance responses, protecting and defending established social bonds. I have recently postulated [36] that these apparently divergent findings might also be reconciled in light of the function of the hypothalamic–pituitary–adrenocortical axis in modulating both social approach and avoidance behaviors [37].

Furthermore, according to an allostatic perspective [38,39,40], the hypothalamus would support the energetic and physiological resources required to dynamically instantiate appropriate and timely socioemotional responses through the monitoring and regulation of bodily signal changes, as well as of hormonal and neuropeptide fluctuations. Such a complex mechanism would possibly imply the instantiation of predictive physiological representations of socioemotional contexts and interactions, and the consecutive evaluation of prediction error signals [41,42,43], to ultimately optimize adaptive anticipatory responses. Indeed, recent evidence in nonhuman primates revealed that the lateral hypothalamus can generate fine prediction signals of reward expectation, uncertainty, and predictability during both approaching and avoiding behaviors relative to appetitive and aversive contexts [44]. These findings corroborate the postulated function of the hypothalamus in mediating the predictive processing of biologically salient signals, and conceivably suggest that such a predictive mechanism might also intervene during socioaffective interactions.

Future research exploiting ultra-high-resolution neuroimaging methodologies and advanced methods for measuring neurochemicals and neuropetides is required to better comprehend the complex neurophysiological regulation and neuropeptidergic signaling orchestrated by the hypothalamus during human socioemotional interactions. Moreover, a multimodal integrated approach, linking genetic, or other risk factors for socioemotional disorders, to neurophysiological, neurochemical, or behavioral mechanisms, will also help to clarify the exact role of this brain region in mediating maladaptive interpersonal behaviors observed in several neuropsychiatric disorders with severe socioaffective impairments such as autism, schizophrenia, and antisocial personality disorder.
